# Osteoblasts in a Perfusion Flow Bioreactor—Tissue Engineered Constructs of TiO_2_ Scaffolds and Cells for Improved Clinical Performance

**DOI:** 10.3390/cells11131995

**Published:** 2022-06-22

**Authors:** Maria Schröder, Janne Elin Reseland, Håvard Jostein Haugen

**Affiliations:** Department of Biomaterials, Institute of Clinical Dentistry, University of Oslo, NO-0317 P.O. Box 1109 Blindern Oslo, Norway; maria.schroder@odont.uio.no (M.S.); j.e.reseland@odont.uio.no (J.E.R.)

**Keywords:** perfusion bioreactor, synthetic bone scaffold, wall shear stress, fluid flow, bone tissue engineering, human osteoblasts

## Abstract

Combining biomaterial scaffolds with cells serves as a promising strategy for engineering critical size defects; however, homogenous cellular growth within large scaffolds is challenging. Mechanical stimuli can enhance bone regeneration by modulating cellular growth and differentiation. Here, we compare dynamic seeding in a perfusion flow bioreactor with static seeding for a synthetic bone scaffold for up to 21 days using the cell line MC3T3-E1 and primary human osteoblast, confocal laser scanning microscopy, and real-time reverse transcriptase-polymerase chain reaction. The secretion of bone-related proteins was quantified using multiplex immunoassays. Dynamic culture improved cellular distribution through the TiO_2_ scaffold and induced a five-fold increase in cell number after 21 days. The relative mRNA expression of osteopontin of MC3T3-E1 was 40-fold enhanced after 7 and 21 days at a flow rate of 0.08 mL/min, and that of collagen type I alpha I expression was 18-fold after 21 days. A flow rate of 0.16 mL/min was 10-fold less effective. Dynamic culture increased the levels of dickkopf-related protein 1 (60-fold), osteoprotegrin (29-fold), interleukin-6 (23-fold), interleukin-8 (36-fold), monocyte chemoattractant protein 1 (28-fold) and vascular endothelial growth factor (6-fold) in the medium of primary human osteoblasts after 21 days compared to static seeding. The proposed method may have clinical potential for bone tissue engineering.

## 1. Introduction

Today only a handful of scaffolds lead the craniomaxillofacial market. As a result of increased regulatory restrictions, particularly in Europe (European Tissue and Cells Directive; EUTCD, 2004), and the Medical Device Regulation (MDR, 2017), allografts are less used in clinical practice, and their suppliers may be limited in the future [1,2]. European dentists still prefer xenografts as bone scaffolds. However, under MDR, xenografts experience a more challenging regulatory pathway [3]. More scientific literature and comparative studies are needed to convince dentists that new bone grafts (BGs) can provide more advantages than current xenografts. This may provide a shift from allografts and xenografts to synthetic scaffold BG substitutes [4]. One example of a synthetic scaffold is titanium dioxide (TiO_2_), a block ceramic BG substitute with porous architecture which allows the formation of bone and vascularization, and has compressive strength similar to trabecular bone and biocompatible properties [5,6]. The ability of these scaffolds to promote the attachment, growth and differentiation of osteoblastic cells was demonstrated in vitro [7]. In addition, previous studies showed both bone ingrowth and angiogenesis within the structures of the TiO_2_ block in vivo minipig and dog models [6,8]. These scaffolds are now in clinical use. Thereon, this is a promising biocompatible material which is also reported to have bioactive properties [9,10].

Although TiO_2_ scaffolds favor new bone ingrowth in vivo [6,8,11], in chronic bone defects the rate of bone growth was found to be too slow [12]. Therefore, bone tissue engineering (BTE) strategies are needed for these TiO_2_ scaffolds to increase clinical translation. One strategy may be to harvest osteoprogenitor/osteogenic cells from the patient and combine these with the scaffold in a dynamic culturing system.

Various dynamic culturing systems, including spinner flasks [13], rotating wall vessels [13,14], and perfusion bioreactors [15,16,17] were applied to BTE to overcome diffusion limitations of nutrients and oxygen in static culture environments. These cause reduced cellular viability and growth in the scaffold center compared to its periphery, limiting the ingrowth of new tissue into the scaffold [18,19,20]. Among these systems, perfusion bioreactors are most advantageous, as they provide enhanced delivery of nutrients and metabolites as well as the removal of waste products throughout the entire porous scaffold [21]. In addition to enhanced mass transport, fluid flow provides mechanical stimulation to the cells, similar to the bone mechanical environment in vivo. Here, mechanical loading exposes bone cells to mechanical stretch and interstitial fluid flow within the porous lacuna-canalicular bone network [22,23]. Fluid flow through a bone scaffold enhances osteoprogenitor cells’ differentiation and improves osteoblast function [15,24,25]. However, the fluid flow and its wall shear stress (WSS) depend on scaffold pore structure and morphology. One cannot translate inlet fluid flow from one scaffold system to another without prior examination of computational fluid dynamics (CFD).

We analyzed the fluid flow and stimuli acting on cells in porous TiO_2_ scaffolds using CFD under simulated perfusion culture conditions [26]. However, the prolonged effect of fluid flow and shear stress stimuli on bone cells, and their growth and differentiation in the scaffolds under experimental conditions, have not yet been studied.

This study investigated whether the in silico modeling for TiO_2_ scaffolds can be verified experimentally using a custom-made flow perfusion bioreactor, and whether the system can be a beneficial BTE strategy for the scaffolds. The mouse preosteoblastic cell line MC3T3-E1 represents a standard model to investigate the effect of mechanical stimuli in three-dimensional bone scaffolds; fewer studies use human mesenchymal stem cells or osteoblasts. However, interspecies differences hamper the transfer of the results to clinical use [27].

We aimed to:

(1) Validate whether perfusion seeding of TiO_2_ scaffold versus static seeding is beneficial for bone scaffolds prior to clinical use based on in silico modeling.
(2) Analyze the effect of shear fluid flow on the growth and differentiation of MC3T3-E1 cells and human osteoblasts cultured on TiO_2_ scaffolds.
(3) Analyze the validity of the results obtained from the cell line osteoblasts compared to primary human osteoblasts.

**Figure 1:**
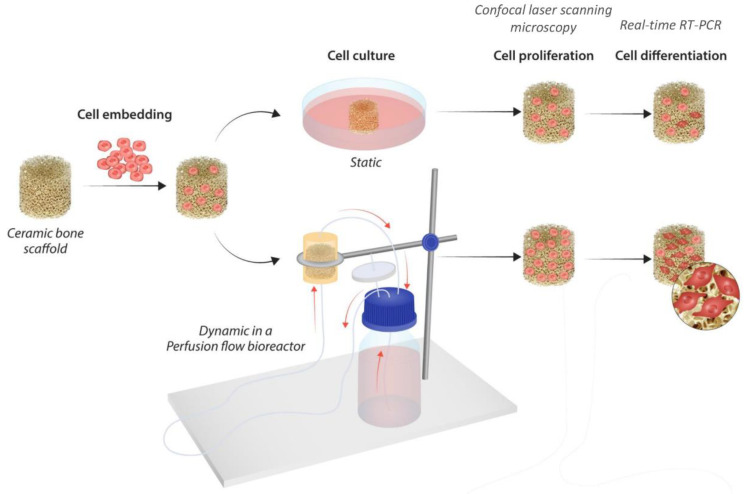
Experimental design of the study.

An overview of the study’s experimental design is shown in Figure 1.

## 2. Materials and Methods

### 2.1. Fabrication of TiO_2_ Scaffolds

Porous TiO_2_ scaffolds, with 8 mm height and 9 mm diameter, were produced using commercial TiO_2_ powder (Kronos 1171, Kronos Titan GmbH, Leverkusen, Germany) and polymer sponge replication as previously described [28]. In brief, cylindrical polyurethane foam templates were coated with TiO_2_ slurry, dried, the polymer template was burned out, and the scaffolds were sintered at 1500 °C for 40 h. The scaffolds were steam sterilized at 121 °C for 20 min before cell culture.

### 2.2. Cell Culture and Seeding

The mouse preosteoblastic cell line MC3T3-E1 (ATCC, Manassas, VA, USA) was cultured in α-MEM + GlutaMAX (Gibco) supplemented with 10% fetal bovine serum (FBS, PAA Laboratories GmbH, Pasching, Austria), 100 U mL^−1^ penicillin, 100 µg mL^−1^ streptomycin (Gibco) and primary human osteoblasts (Cambrex Bio Science, Walkersville, MD, USA) from two donors (one from humerus and one from tibia) in osteoblast growth medium supplemented with 10% FBS, 0.1% gentamicin sulphate/amphotericin B and 0.1% ascorbic acid (Lonza, Walkersville, MD, USA) at 37 °C in a humidified atmosphere of 95% air and 5% CO_2_. As previously described [8], an agitated seeding method was used to ensure homogenous cell distribution throughout the scaffold. In brief, scaffolds were placed in non-adherent 48 well plates, a solution of 2 × 10^5^ cells in culture medium was pipetted against the wall of each sample well, and the plates were agitated for 3 h at 37 °C. The scaffolds were then moved to a new 48 well plate and incubated for 24 h at 37 °C in the corresponding culture medium.

### 2.3. Perfusion Bioreactor System and Flow Culture

The flow perfusion bioreactor system consisted of a vertical cylindrical polyether ether ketone chamber with a 6 cm height and a 3 cm diameter with a round recess (diameter: 0.9 cm) for the scaffolds, which was connected to a 200 mL medium reservoir bottle with a customized cap of platinum-coated silicon tubing (HECO–Laboratorieutstyr AS, Oslo, Norway). Sterile gas exchange was maintained through a disposable air filter (0.2 µm PES, VWR) on the medium reservoir. Medium flow from the reservoir to the perfusion chamber and back was generated using a peristaltic roller pump (Masterflex, Cole-Parmer, Vernon Hills, IL, USA). A small segment of the flow circuit consisted of Masterflex PharMed BPT tubing (Cole-Parmer, Vernon Hills, IL, USA) to ensure sufficient mechanical resistance of the tubing in the peristaltic pump. All components were autoclaved at 121 °C for 20 min and assembled under sterile conditions in a laminar flow hood. Each perfusion chamber housed two scaffolds. To allow for a homogenous distribution of medium flow through the seeded constructs, a grid (polytetrafluoroethylene; diameter: 0.9 cm; hole diameter: 0.1 cm) was placed in front of each scaffold. The perfusion chamber was filled with theculture medium, and the bioreactor system was placed in an incubator and operated at 37 °C. Two continuous media flow rates were analyzed in this study: 0.16 mL/min (inlet fluid velocity: 34 µm/s, based on [26]) and 0.08 mL/min (inlet fluid velocity: 17 µm/s). TiO_2_ scaffolds cultured without flow were placed in a new 48-well plate and incubated at 37 °C in the corresponding culture medium. The media on both flow and non-flow cultures were changed twice a week. At day 7 and 21, the culture media from both flow and non-flow cultures were collected for analysis and scaffolds were harvested. Scaffolds were washed twice in PBS and stored either in this solution at 4 °C until microscopy or frozen at −80 °C until DNA quantification and RT-PCR.

### 2.4. DNA Quantification

TiO_2_ scaffolds were incubated in lysis buffer (10 mM Tris pH 8, 1 mM EDTA, 0.2% *v*/*v* Triton X-100) for 1 h on ice. According to the manufacturer’s protocol, double-stranded DNA (dsDNA) was quantified using Qubit 1X dsDNA HS Assay Kit and Qubit 4.0 Fluorometer (Thermo Fisher Scientific, Waltham, MA, USA).

### 2.5. Confocal Laser Scanning Microscopy

TiO_2_ scaffolds were washed twice in PBS, fixed in 4% paraformaldehyde and washed again in PBS. Cells were permeabilized with 0.1% triton X-100 for 5 min, washed in PBS and blocked with 1% BSA in PBS for 60 min at room temperature. Following this, cells were stained with Alexa Fluor 568 Phalloidin (Thermo Fisher Scientific, Waltham, MA, USA) at 1:400 dilution in 1% BSA for 60 min and washed twice in PBS. Cells were counterstained with Hoechst 33342 (1 µg/mL in PBS; Sigma-Aldrich, Saint Louis, MO, USA), and scaffolds were stored in this solution until microscopy. Prior to imaging, the scaffolds were halved with a scalpel. Imaging was performed using a Leica SP8 confocal microscope (Leica Microsystems CMS GmbH, Mannheim, Germany) using 405- and 552-nm excitation and 420- to 480-nm and 580- to 630-nm emission filters for Hoechst 33342 and Alexa Fluor 568, respectively. Confocal images were processed using ImageJ software (NIH, Bethesda, MD, USA).

### 2.6. RNA Isolation and Real-Time RT-PCR Analysis

Total RNA from TiO_2_ scaffolds was isolated using RNeasy mini kit (Qiagen, Hilden, Germany) with some modifications to the manufacturer’s protocol. In brief, scaffolds were incubated in a lysis buffer for 1 h at 4 °C and agitated on an orbital shaker for 10 min. Then the lysate was sonicated (UP50H; Hielscher Ultrasonics GmbH, Teltow, Germany) for 10 s. The rest of the isolation followed the manufacturer’s protocol. cDNA was synthesized using RevertAid First Strand cDNA Synthesis Kit and oligo dT primers according to the manufacturer’s protocol (Thermo Fisher Scientific, Waltham, MA, USA). Real-time PCR was carried out in BioRad CFX Connect System (Bio-Rad Laboratories, Hercules, CA, USA), using SYBR green-based assay iQ SYBR supermix (Bio-Rad Laboratories, Hercules, CA, USA). Real-time RT-PCR data were analyzed using the 2^−ΔΔCt^ method 2 (Delta Delta C(T)) [29]. The primer sequences are listed in Table 1.

### 2.7. Quantification of Proteins Secreted in the Cell Culture Medium

Multianalyte profiling of protein levels in the culture media was performed on the Luminex 200 system employing xMAP technology (Luminex Corp., Austin, TX, USA). Acquired fluorescence data were analyzed using xPONENT 3.1 software (Luminex, Austin, TX, USA). The secretion of dickkopf-related protein 1 (DKK-1), osteoprotegerin (OPG), interleukin-6 (IL-6), interleukin-8 (IL-8), monocyte chemoattractant protein 1 (MCP1) and vascular endothelial growth factor (VEGF) to the culture medium were measured using the Milliplex Human Bone Panel and a Cytokine/Chemokine kit. For the analysis, aliqouts of the culture media from the respective groups, the static samples and perfused samples, were pooled. The culture media from the perfused samples were concentrated five- to eight-fold using MicrosepTM centrifugal tubes with a 3 kDa cut-off. All further analyses were performed according to the manufacturer’s protocols.

### 2.8. Statistical Analysis

Statistical analysis was performed using SigmaPlot software version 14.0 (Systat Software, San Jose, CA, USA). Data obtained using DNA assay and real-time RT-PCR (ΔΔCt values) were compared between the groups using a *t*-test or a Mann–Whitney U test, depending on their normal distribution. Data obtained using Luminex assay were compared between static and perfused samples using a Holm–Sidak test. A probability of ≤0.05 was considered significant.

## 3. Results

### 3.1. Effect of Medium Flow and Influence of Flow Rate on Growth and Distribution of MC3T3-E1 Cells Cultured on TiO_2_ Scaffolds

**Figure 2:**
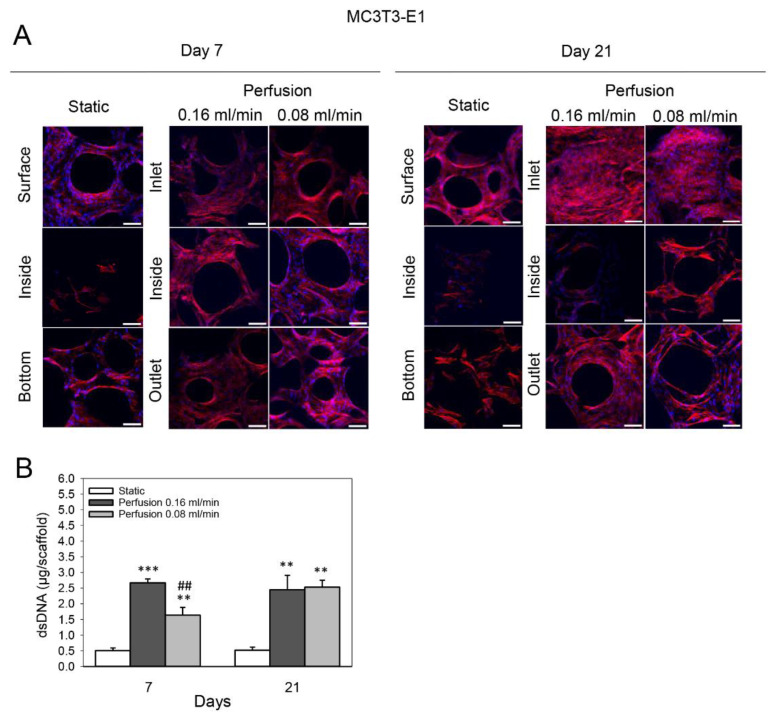
Effect of medium flow and influence of flow rate on growth and distribution of MC3T3-E1 cells cultured on TiO_2_ scaffolds without flow (static) and with continuous flow (flow rates of 0.16 mL /min and 0.08 mL/min) for 7 and 21 days. (**A**) Immunofluorescence images of F-actin (red) and cell nuclei (blue). The top and bottom refer to the scaffold surfaces exposed to the medium and touching the well plate, respectively. Inlet and outlet refer to scaffold surfaces from which the flow entered and exited, respectively. Scaffolds were cut with a scalpel to observe the cellular distribution inside the scaffold. Scalebar = 100 µm. (**B**) Cultured scaffolds’ double-stranded DNA (dsDNA) (shown in µg/scaffold) was quantified. Values represent the mean ± SD. Significant differences were analyzed with a SigmaPlot *t*-test. ** *p* < 0.01 and *** *p* < 0.001 indicate significance compared to the static group; ^##^ *p* < 0.01 indicates significance compared to the perfusion 0.16 mL/min group.

Confocal microscopy revealed an uneven distribution of MC3T3-E1 cells on TiO_2_ scaffolds cultured statically for 7 and 21 days. While the scaffold side (surface) exposed to the medium was highly populated with cells, fewer cells were found inside and on the bottom of the scaffold. Perfusion culture at both the higher (0.16 mL/min) and lower (0.08 mL/min) flow rates resulted in a homogenous cellular distribution throughout the TiO_2_ scaffold after 7 days. After 21 days of perfusion culture at both the higher and lower flow rates, the scaffold side from which the flow entered (inlet) was covered with a dense layer of cells, with most pores completely bridged by the cells. Compared to the inlet side, fewer cells were observed inside and on the side of the scaffold from which the flow exited (outlet). However, perfusion culture still increased the number of cells in these areas compared to the static group (Figure 2A). Confocal microscopy observations resembled the DNA assay. Determination of dsDNA content of the scaffolds showed that perfusion culture increased the proliferation of MC3T3-E1 cells after 7 and 21 days compared to static culture. A flow rate of 0.08 mL/min enhanced the dsDNA content 3.2-fold compared to the static group after 7 days (*p* = 0.002); a flow rate of 0.16 mL/min induced a 5.3-fold increase (*p* < 0.001). The increase at flow rate 0.16 mL/min was significantly higher than at 0.08 mL/min (*p* = 0.001). After 21 days, the dsDNA content of constructs perfused at 0.16 mL/min and 0.08 mL/min increased 4.7-fold and 4.9-fold compared to statically cultured scaffolds (*p* = 0.003 and *p* = 0.004), respectively (Figure 2B).

### 3.2. Effect of Medium Flow on Growth and Distribution of Primary Human Osteoblasts Cultured on TiO_2_ Scaffolds

**Figure 3:**
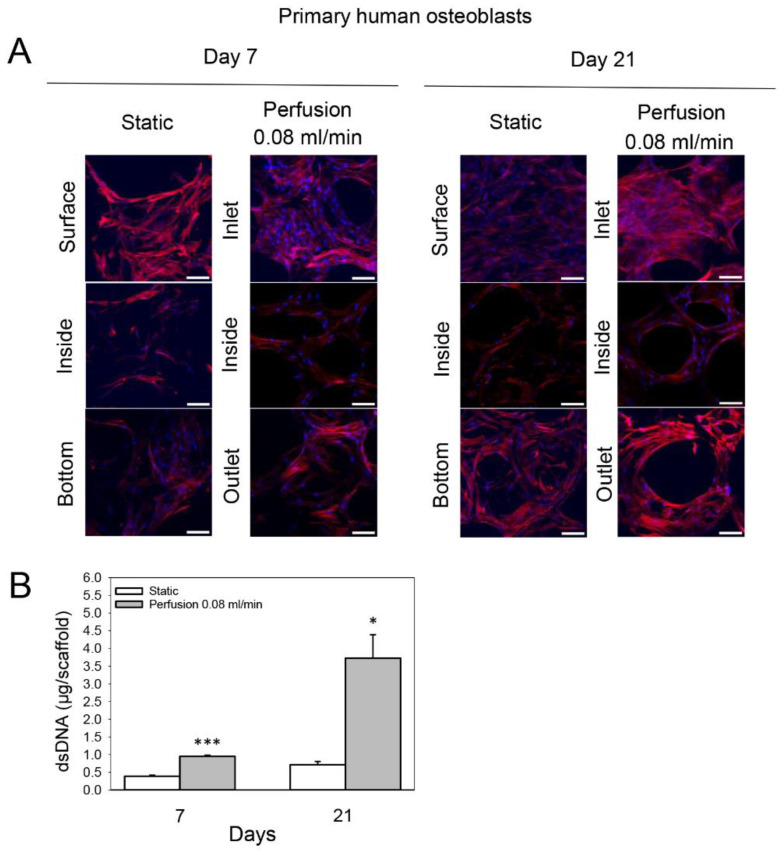
Effect of medium flow on growth and distribution of primary human osteoblasts cultured on TiO_2_ scaffolds without flow (static) and with continuous flow (flow rate of 0.08 mL/min) for 7 and 21 days. (**A**) Immunofluorescence images of F-actin (red) and cell nuclei (blue). The top and bottom refer to the scaffold surfaces exposed to the medium and touching the well plate, respectively. Inlet and outlet refer to scaffold surfaces from which the flow entered and exited, respectively. Scaffolds were cut with a scalpel to observe the cellular distribution inside the scaffold. Scalebar = 100 µm. (**B**) Cultured scaffolds’ double-stranded DNA (dsDNA) (shown in µg/scaffold) was quantified. Values represent the mean ± SD. Significant differences were analyzed with a SigmaPlot *t*-test. * *p* < 0.05 and *** *p* < 0.001 indicate significance compared to static group.

The distribution of primary human osteoblasts on statically cultured TiO2 scaffolds after 7 and 21 days was similar to that of MC3T3-E1 cells. However, more cells were observed inside and at the bottom of the constructs at both time points. Perfusion culture at 0.08 mL/min enhanced cellular growth after 7 and 21 days mainly on the flow inlet side of the scaffolds compared to statically cultured constructs, and slightly more cells were observed inside and at the flow outlet side (Figure 3A). In line with this, the dsDNA content of these scaffolds was 2.5-fold greater compared to the static group on day 7 (*p* < 0.001) and 5.2-fold greater on day 21 (*p* = 0.029) (Figure 3B).

### 3.3. Effect of Medium Flow and Influence of Flow Rate on Osteogenic Gene Expression of MC3T3-E1 Cells Cultured on TiO_2_ Scaffolds

**Figure 4:**
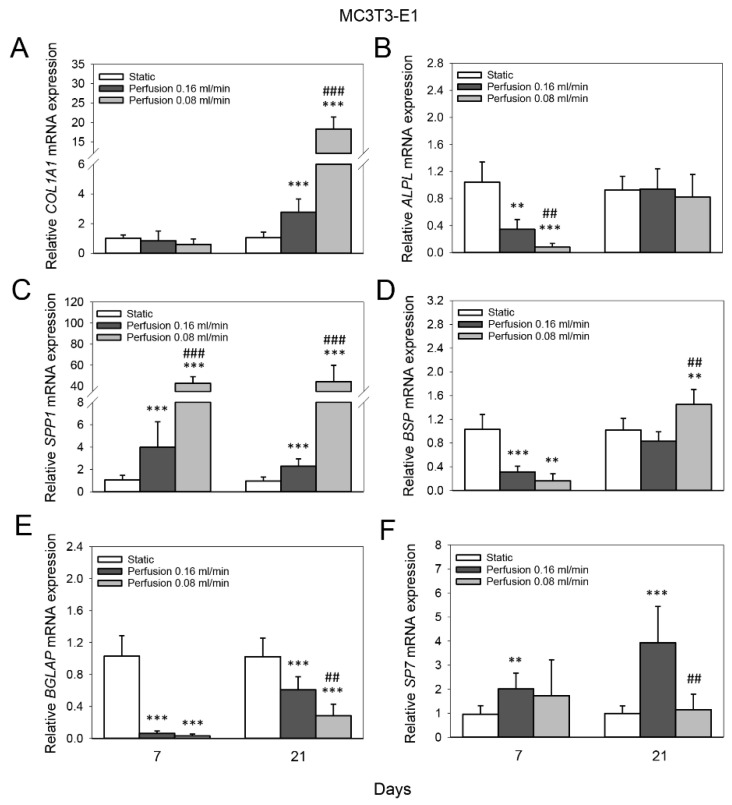
Effect of medium flow and influence of flow rate on osteogenic gene expression of MC3T3-E1 cells cultured on TiO_2_ scaffolds. Relative mRNA levels of (**A**) collagen type I alpha 1 (*COL1A1*), (**B**) alkaline phosphatase (*ALPL*), (**C**) osteopontin (*SPP1*), (**D**) bone sialoprotein (*BSP*), (**E**) osteocalcin (*BGLAP*) and (**F**) osterix (*SP7*) without flow (static) and with continuous flow (flow rates of 0.16 mL /min and 0.08 mL/min) at day 7 and 21. Data represent fold changes of target genes normalized to reference gene *GAPDH* (glyceraldehyde-3-phosphate dehydrogenase). Values represent the mean ± SD. Significant differences were analyzed using a SigmaPlot *t*-test. ** *p* < 0.01 and *** *p* < 0.001 indicate significance compared to the static group; ^##^ *p* < 0.01 and ^###^ *p* < 0.001 indicate significance compared to perfusion 0.16 mL/min group.

Real-time RT-PCR analysis revealed that both the higher (0.16 mL/min) and lower (0.08 mL/min) flow rates enhanced the mRNA expression of *COL1A1* of MC3T3-E1 cells after 21 days compared to the static group (*p* < 0.001 for both; 2.8-fold and 18.3-fold, respectively). The increase at flow rate 0.08 mL/min was significantly higher than at 0.16 mL/min (*p* < 0.001) (Figure 4A). The mRNA expression of *ALPL* of MC3T3-E1 cells was 3-fold reduced at flow rate 0.16 mL/min after 7 days compared to the static group (*p* = 0.004), and even more so at flow rate 0.08 mL/min (12.8-fold, *p* < 0.001) (0.16 mL/min vs. 0.08 mL/min, *p* = 0.002) (Figure 4B). Moreover, perfusion culture elevated the mRNA expression of *SPP1* of MC3T3-E1 cells after 7 and 21 days compared to static culture. At day 7, flow rate 0.16 mL/min induced a 3.9-fold increase compared to the static group, and flow rate 0.08 mL/min induced an even higher increase (42.7-fold, *p* < 0.001 for both) (0.16 mL/min vs. 0.08 mL/min, *p* < 0.001); a similar pattern was observed after 21 days (Figure 4C). In addition, perfusion culture reduced the mRNA expression of *BSP* of MC3T3-E1 cells after 7 days compared to static culture (flow rate 0.16 mL/min, 3.3-fold (*p* < 0.001), flow rate 0.08 mL/min, 6.3-fold); however, no significant difference was found between the two groups (*p* = 0.055). After 21 days, the lower flow rate induced a slight increase in the mRNA expression of *BSP* (1.4-fold, *p* = 0.003) (Figure 4D). The mRNA expression of *BGLAP* of MC3T3-E1 cells was reduced 16.4-fold at flow rate 0.16 mL/min after 7 days compared to the static group, and 32.8-fold at flow rate 0.08 mL/min (*p* < 0.001 for both); however, no significant difference was found between the two groups (*p* = 0.061). After 21 days, the higher flow rate induced a 1.7-fold reduction in the mRNA expression of *BGLAP* and the lower flow rate induced an even greater reduction (3.6-fold, *p* < 0.001 for both) (0.16 mL/min vs. 0.08 mL/min, *p* = 0.004) (Figure 4E). The mRNA expression of *SP7* of MC3T3-E1 cells was 2-fold enhanced at flow rate 0.16 mL/min after 7 days compared to the static group (*p* = 0.004), and 3.9-fold after 21 days (*p* < 0.001) (Figure 4F).

### 3.4. Effect of Medium Flow on Osteogenic Gene Expression of Primary Human Osteoblasts Cultured on TiO_2_ Scaffolds

**Figure 5:**
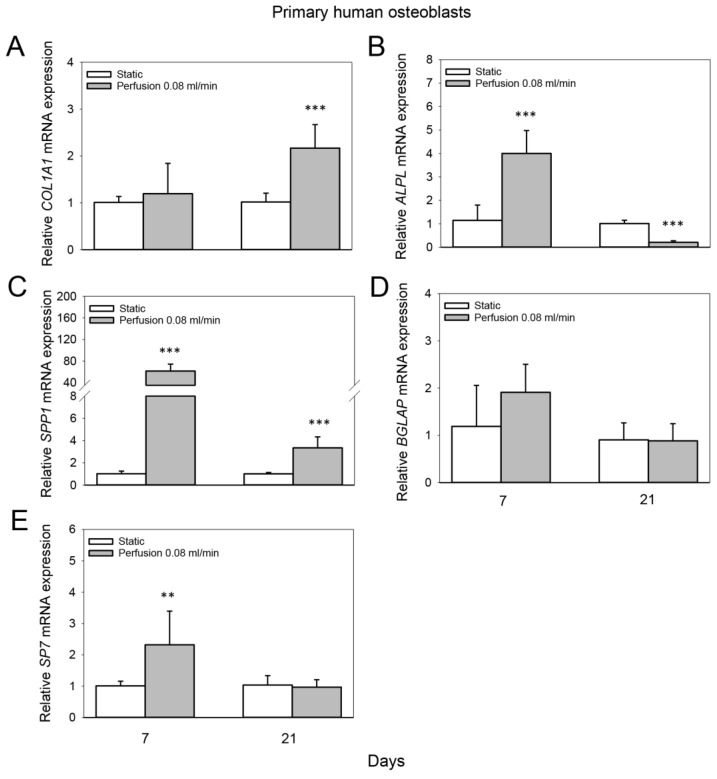
Effect of medium flow on osteogenic gene expression of primary human osteoblasts cultured on TiO_2_ scaffolds. Relative mRNA levels of (**A**) collagen type I alpha 1 (*COL1A1*), (**B**) alkaline phosphatase (*ALPL*), (**C**) osteopontin (*SPP1*), (**D**) osteocalcin (*BGLAP*) and (**E**) osterix (*SP7*) without flow (static) and with continuous flow (flow rate of 0.08 mL/min) at day 7 and 21. Data represent fold changes of target genes normalized to reference gene *GAPDH* (glyceraldehyde-3-phosphate dehydrogenase). Values represent the mean ± SD. Significant differences were analyzed using a SigmaPlot *t*-test. ** *p* < 0.01 and *** *p* < 0.001 indicate significance compared to static group.

Perfusion culture at 0.08 mL/min induced a 2.2-fold increase in the mRNA expression of *COL1A1* of primary human osteoblasts after 21 days compared to the static group (*p* < 0.001) (Figure 5A). The mRNA expression of *ALPL* of primary human osteoblasts was 4-fold enhanced at a flow rate of 0.08 mL/min after 7 days compared to static culture (*p* < 0.001), while after 21 days, a 4.9-fold reduction was observed (*p* < 0.001) (Figure 5B). Furthermore, *SPP1* mRNA expression of primary human osteoblasts was elevated 61.5-fold at a flow rate of 0.08 mL/min after 7 days compared to static culture and 3.3-fold after 21 days (*p* < 0.001 for both) (Figure 5C). The mRNA expression of *BGLAP* of primary human osteoblasts on TiO_2_ scaffolds was not significantly altered after 7 or 21 days by perfusion culture at 0.08 mL/min compared to the static group (Figure 5D). However, the mRNA expression of *SP7* was 2.3-fold enhanced at a flow rate of 0.08 mL/min after 7 days compared to static culture (*p* = 0.013) (Figure 5E).

### 3.5. Effect of Medium Flow on the Secretion of Bone-Related Proteins from Primary Human Osteoblasts Cultured on TiO_2_ Scaffolds

**Figure 6:**
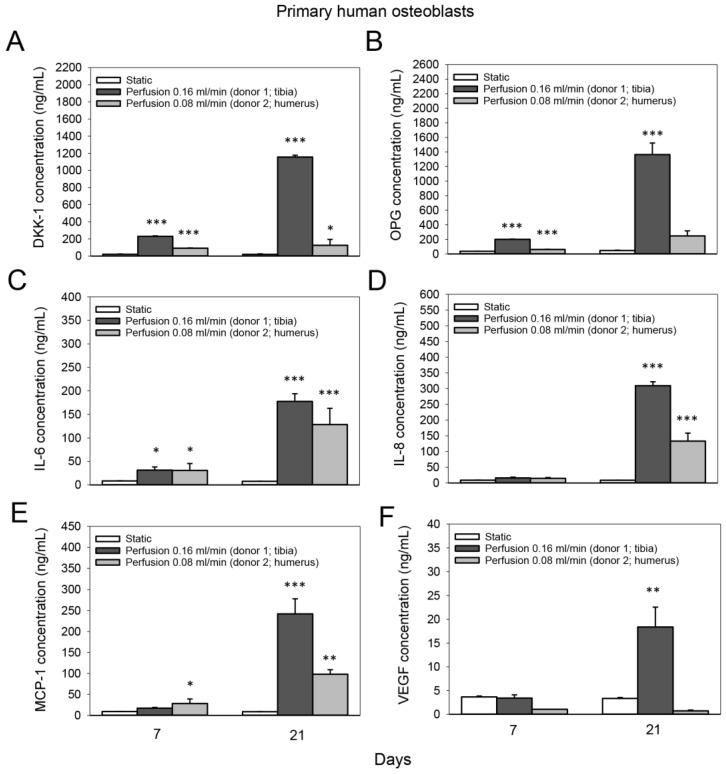
Effect of medium flow on the secretion of bone-related proteins from primary human osteoblasts cultured on TiO_2_ scaffolds. Concentration (in ng/mL) of (**A**) dickkopf-related protein 1 (DKK-1), (**B**) osteoprotegrin (OPG), (**C**) interleukin-6 (IL-6), (**D**) interleukin-8 (IL-8), (**E**) monocyte chemoattractant protein 1 (MCP-1) and **(F)** vascular endothelial growth factor (VEGF) in the culture media of two different human osteoblasts donors without flow (static) and with continuous flow. The culture media of the static and perfused samples were pooled from two scaffolds. A flow rate of 0.16 mL/min was conducted with donor 1 (isolated from the tibia), and a flow rate of 0.08 mL/min was conducted with donor 2 (isolated from the humerus). Values represent the mean ± SD. Significant differences were analyzed using a SigmaPlot Holm-Sidak test. * *p* < 0.05, ** *p* < 0.01 and *** *p* < 0.001 indicate significance compared to staticgroup. Statistical comparison between the two perfusion groups was not performed due to the use of two different osteoblast donors.

The concentration of DKK-1 in the culture medium of primary human osteoblasts increased in the perfusion group after 7 days compared to the static group (donor 1: 10.7-fold; donor 2: 4.3-fold; *p* < 0.001 for both) and after 21 days (donor 1: 60-fold, *p* < 0.001; donor 2: 6.5-fold, *p* = 0.013) (Figure 6A). Moreover, perfusion culture induced 5.5-fold and 29.3-fold increases in OPG levels in the culture medium of primary human osteoblast donor 1 compared to static culture after 7 and 21 days (*p* < 0.001 for both), respectively (Figure 6B). The concentration of IL-6 in the culture medium of primary human osteoblasts increased 3.7-fold in the perfusion group after 7 days compared to the static group (donor 1, *p* = 0.042; donor 2, *p* = 0.03); after 21 days, 23.4-fold and 17-fold increases were observed for donor 1 and donor 2 (*p* < 0.001), respectively (Figure 6C). In line with this, IL-8 levels in the culture medium of primary human osteoblasts increased 35.8-fold (donor 1) and 15.4-fold (donor 2) after 21 days in the perfusion group compared to the static group (*p* < 0.001 for both) (Figure 6D). Furthermore, perfusion culture induced 27.9-fold (donor 1, *p* < 0.001) and 11.3-fold (donor 2, *p* = 0.002) increases in the amount of MCP-1 in the culture medium after 21 days compared to static culture (Figure 6E), and a 5.5-fold rise (donor 1; p=0.001) in VEGF (Figure 6F).

**Figure 7:**
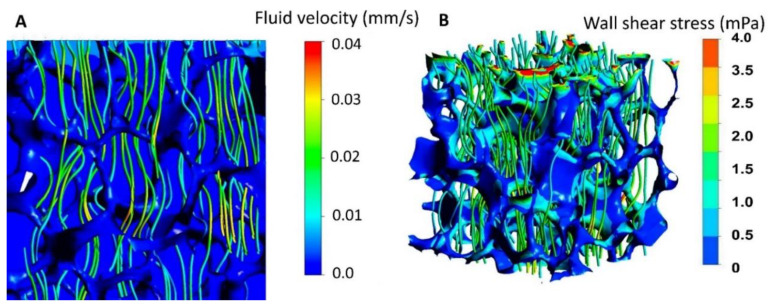
**(A)** The cross-sectional view of static pressure on the walls combined with streamlines, color coded according to velocity magnitude, when the inlet velocity was 0.08 mL/min. **(B)** Wall shear stress distribution in combination with streamlines, color coded according to velocity magnitude, when the inlet velocity was 34 μm/s. Reprinted with permission from Copyright Clearance Center’s RightsLink^®^ service reference number 5302731494559 [26].

The fluid flow and induced WSS can be viewed in Figure 7 based on previously performed CFDs.

## 4. Discussion

The BTE approach using in vitro expansion of cells on a scaffold prior to grafting into bone represents a promising alternative to current clinical treatments. TiO_2_ scaffolds, as used here, show promising results in various in vivo experiments [6,8,11]. However, in a recent study [12] using a chronic non-contained bone defects application, less bone formation was observed in the TiO_2_ groups compared to membrane alone at the final time point of 12 weeks of healing. To improve the clinical performance of bone scaffolds, various bioreactor systems are suggested, including spinner flasks, rotating wall bioreactors, and perfusion systems [21]. Perfusion systems expose cells to shear stress and more efficiently enhance nutrient transfer than other systems [30]; for this reason it was chosen as the bioreactor in this study. Shear influences osteoblastic differentiation [31,32,33,34,35,36], but the magnitude of shear stress cells are exposed to in various bioreactor systems is not always known. Modeling can be a tool for calculating the magnitude of shear stress and yield information on an ideal inlet fluid velocity. Although such modeling for the TiO_2_ scaffolds was performed, it was never validated with in vitro experiments; nevertheless, the inlet fluid velocities were chosen as suggested in Zhang et al.’s simulation [26]. To validate the inlet fluid velocity, we exposed both human and cell line osteoblasts to shear stress to provide insight into the bone cell behavior pathways affected by long-term shear stress. Determining this effect will allow for a greater understanding of perfusion systems, lead to more effective optimization of these systems and potentially lead to a more effective introduction of these systems into clinical BTE.

The ability of material exchange can be characterized by interconnectivity and permeability in a bone scaffold. The predicted permeability of these scaffolds [26] was found to be within the range of cancellous bone by comparative analysis, and should positively influence the cell migration rate [37] when compared to static seeding. The present study clearly illustrates this effect, as significantly more bone cells were found after days 7 and 21. Our findings are comparable to other similar studies [38,39].

TiO_2_ has higher pore interconnectivity and higher permeability (1.678 × 10^−9^ m^2^) than other commercial scaffolds, which was more conducive to nutrient transport and metabolic product excretion and improved in vivo bone ingrowth [40,41]. Variations in inlet fluid velocity and fluid viscosity produce proportional and independent changes in fluid velocity, fluid shear stress and fluid pressure. These variations, here with two different inlet velocities, should cause different levels of mechanical stimuli within the scaffold [42,43]. According to Sandino et al. and Cartmell et al., 37–46 mPa shear stress can stimulate osteoblast differentiation into bone cells [39,44]. Furthermore, differentiation of the cells adhered to the surface wall of the TiO_2_ scaffold should occur with the inlet fluid flow used, as it will provide a WSS from 1.35 to 2.55 mPa [44,45]. Indeed, the osteogenic gene expression of both the cell line and human osteoblasts were altered with the applied fluid flow. For instance, a flow rate of 0.08 mL/min highly upregulated collagen type I and osteopontin gene expression in MC3T3-E1 cells. This is in agreement with similar studies [46,47,48]. The phosphorylated glycoprotein osteopontin is an important factor in the formation of bone. It is secreted by osteoblasts during the early stage of bone development and binds to hydroxyapatite to promote the cell attachment and spreading necessary for bone formation and mineralization [49]. Since a flow rate of 0.16 mL/min was 10-fold less effective in upregulating osteopontin and collagen type I gene expression of the MC3T3-E1, we concentrated our efforts for the primary human osteoblasts on a flow rate of 0.08 mL/min. Here, we also observed a strong effect of fluid flow on the gene expression of osteopontin after 7 days; additionally, the gene expression of alkaline phosphatase, a crucial enzyme in the initiation of the bone mineralization process [50], was moderately enhanced. It slightly increased osterix expression, a transcription factor involved in osteoblast differentiation and bone formation [51], after 7 days. This is in contrast to the cell line osteoblasts and may be explained by interspecies differences in cellular response to fluid flow [52], highlighting the importance of validating the obtained results from the standardized osteoblast model in this study with primary human cells to improve the clinical performance of the TiO_2_ scaffolds. Another reason for the discrepant results may be differences in the osteogenic differentiation stage of the two cell types during the flow culture [53]. In line with this, we observed that the gene expression of alkaline phosphatase, bone sialoprotein and osteocalcin (proteins involved in early and late osteogenic differentiation [54]) in cell line osteoblasts was remarkably reduced after 7 days of flow culture, while the expression of osteopontin was upregulated after 7 and 21 days. Osteopontin is expressed during two stages of osteogenesis, the early proliferative stage and prior to mineralization [54]. Hence, it is reasonable that human osteoblasts were at a more advanced stage of osteogenic differentiation than the cell line osteoblasts during the flow culture. However, using a culture medium specific to each cell type during the long-term perfusion may have also influenced the osteogenic gene expression.

We also aimed to analyze how applied shear stress affects the communication of primary human osteoblasts to the bone microenvironment. We observed that fluid flow increased the release of DKK-1, OPG, IL-6 and IL-8, factors modulating bone remodeling [55,56,57,58] from the human osteoblasts. In addition, fluid flow enhanced the release of the angiogenic factor MCP-1, which stimulates angiogenesis by upregulating VEGF [59], a key factor in promoting vascular growth during bone regeneration. VEGF is also involved in the coupling of angiogenesis and osteogenesis [60]. This may further promote vascularization in the TiO_2_ scaffold interior during bone regeneration [6].

This study analyzed the effect of long-term shear stress on osteoblasts in the porous TiO_2_ scaffolds. Several studies analyze the short-term responses of osteoblasts to fluid flow in bone scaffolds [44,61,62]. However, these are less suitable for BTE approaches, as they cannot provide insights into essential stages during bone formation, such as the late osteogenic differentiation of osteoprogenitor cells, collagen matrix deposition or mineralization. For a BTE approach, it is vital to carefully study the long-term effect of shear stress on cells inside a bone scaffold to determine the ideal bioreactor culture duration prior to implantation. Furthermore, it has to be considered that the flow profile will change with time as the cells proliferate and start to deposit matrix.

## 5. Conclusions

In this study, we verified in silico modeling with an experimental approach using a custom-made perfusion flow bioreactor system and a synthetic bone graft substitute that is in clinical use. We used a standardized osteoblast model to investigate the effect of fluid flow generated by the system on the cellular response and were able to validate the results with primary human osteoblasts. We show that the perfusion system with the examined TiO_2_ bone scaffolds had a positive effect on cellular growth and distribution. In addition, the gene expression of osteopontin and collagen type I alpha I was upregulated by the applied fluid flow, suggesting an effect on osteogenic differentiation. Primary human osteoblasts cultured in the flow bioreactor system communicated to the bone microenvironment by an increase in factors related to bone remodeling and angiogenesis. The proposed method may facilitate an increase in the clinical performance of synthetic bone scaffolds.

## Figures and Tables

**Table 1:** Primer sequences used for real-time RT-PCR analysis.

Protein	Gene	Primer Sequence (5′–3′)
Mouse glyceraldehyde-3-phosphate dehydrogenase	m-*GAPDH* m-*GAPDH*	f ACCCAGAAGACTGTGGATGG r CACATTGGG-GGTAGGAACAC
Mouse collagen type I alpha 1	m-*COL1A1* m-*COL1A1*	f AGAGC-ATGACCGATGGATTC r CCTTCTTGAGGTTGCCAGTC
Mouse alkaline phosphatase	m-*ALPL* m-*ALPL*	f AACCCAGACACAAGCATTCC r GAGAGCGAAGGGTC-AGTCAG
Mouse bone sialoprotein	m-*BSP* m-*BSP*	f GAAA-ATGGAGACGGCGATAG r ACCCGAGAGTGTGGAAAGTG
Mouse osteopontin	m-*SPP1* m-*SPP1*	f TCTGCGGCAGGCATTCTCGG r GTCA-CTTTCACCGGGAGGGAGGA
Mouse osteocalcin	m-*BGLAP* m-*BGLAP*	f CCGGGAGCAG-TGTGAGCTTA r TAGATGC-GTTTGTAGGCGGTC
Mouse osterix	m-*SP7* m-*SP7*	f AC-TGGCTAGGTGGTGGTCAG r GGTAGGGAGC-TGGGTTAAGG
Human-glyceraldehyde-3-phos-phate dehydrogenase	h-*GAPDH* h-*GAPDH*	f CTCTGCTCCTCCTGTTCGAC r ACGACCAAATCCGTTGACTC
Human collagen type I alpha 1	h-*COL1A1* h-*COL1A1*	f CCAAATCCG-ATGTTTCTGCT r CATCTCCCCTTCGTTTTTGA
Human alkaline phosphatase	h-*ALPL* h-*ALPL*	f AGACGCGCCTGGTAGTTGT r GACAAGAAGCCCTTCACTGC
Human osteopontin	h-*SPP1* h-*SPP1*	f TGAGGTGATGTCCTCGTCTG r GCC-GAGGTGATAGTGTGGTT
Human osteocalcin	h-*BGLAP* h-*BGLAP*	f GCTTCACCCTCGAAATGGTA r GCAAGTAGCGCCAATCTAGG
Human osterix	h-*SP7* h-*SP7*	f TACCCC-ATCTCCCTTGACTG r GCTGCAAGCTCTCCATAACC

## Data Availability

Data are available upon request from the corresponding author.
